# 2,5-Di­bromo-3,6-dimeth­oxycyclo­hexa-2,5-diene-1,4-dione

**DOI:** 10.1107/S1600536814011787

**Published:** 2014-05-24

**Authors:** Ersin Orhan, Amine Garci, Bruno Therrien

**Affiliations:** a Institut de Chimie, Université de Neuchâtel, Avenue de Bellevaux 51, CH-2000 Neuchâtel, Switzerland

## Abstract

In the structure of the title compound, C_8_H_6_Br_2_O_4_, the complete mol­ecule is generated by the application of a centre of inversion. The mol­ecule is planar (r.m.s. deviation for all non-H atoms but methyl C = 0.0358 Å), with only the methyl groups being deviated from the plane [by ±0.321 (4) Å]. In the crystal packing, Br⋯O(methoxy) halogen bonds [3.2407 (19) Å] connect molecules into supramolecular layers parallel to (101).

## Related literature   

For the synthesis of the title compound, see: Viault *et al.* (2011 ▶). For the structure of bromanilic acid, see: Robl (1987 ▶). For similar structures with a 2,5-cyclo­hexa­diene-1,4-dione core, see: Nakatsuji *et al.* (2009 ▶). For an article dealing with the biological relevance of this type of compound, see: Viault *et al.* (2013 ▶). For papers using the title compound as a synthetic precursor, see: Khan & Driscoll (1976 ▶); Tatsuta *et al.* (2001 ▶); Kasahara & Kondo (2006 ▶); Gan *et al.* (2009 ▶). For metalla-assemblies obtained with analogous building blocks, see: Gupta *et al.* (2014 ▶); Therrien (2009 ▶).
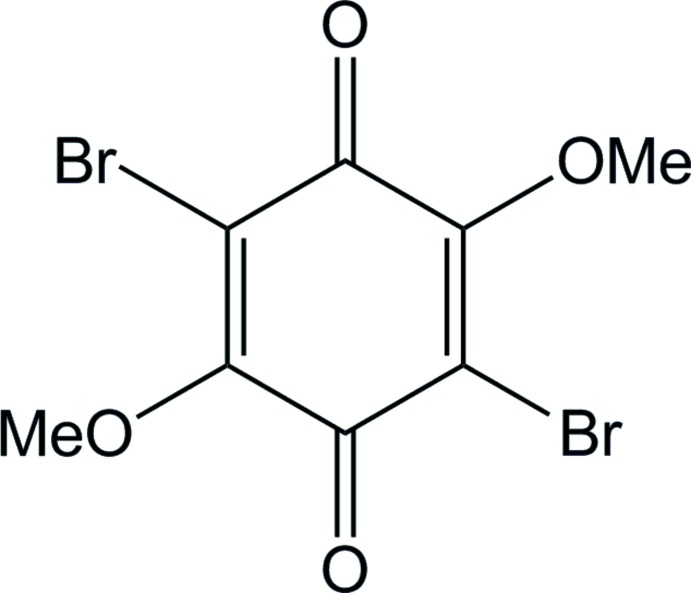


## Experimental   

### 

#### Crystal data  

C_8_H_6_Br_2_O_4_
*M* *_r_* = 325.95
Monoclinic, 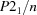

*a* = 9.4456 (9) Å
*b* = 5.4877 (3) Å
*c* = 10.0341 (9) Å
β = 113.846 (7)°
*V* = 475.71 (7) Å^3^
*Z* = 2
Mo *K*α radiation
μ = 8.50 mm^−1^
*T* = 173 K
0.23 × 0.21 × 0.20 mm

#### Data collection  

Stoe IPDS diffractometer
Absorption correction: part of the refinement model (Δ*F*) (*DIFABS*; Walker & Stuart, 1983 ▶) *T*_min_ = 0.246, *T*_max_ = 0.704
8772 measured reflections
1284 independent reflections
1144 reflections with *I* > 2σ(*I*)
*R*_int_ = 0.071

#### Refinement  

*R*[*F* ^2^ > 2σ(*F* ^2^)] = 0.028
*wR*(*F* ^2^) = 0.067
*S* = 1.04
1284 reflections
65 parameters
H-atom parameters constrained
Δρ_max_ = 0.86 e Å^−3^
Δρ_min_ = −0.98 e Å^−3^

### 

Data collection: *EXPOSE* (Stoe & Cie, 2000 ▶); cell refinement: *CELL* (Stoe & Cie, 2000 ▶); data reduction: *INTEGRATE* (Stoe & Cie, 2000 ▶); program(s) used to solve structure: *SHELXS97* (Sheldrick, 2008 ▶); program(s) used to refine structure: *SHELXL97* (Sheldrick, 2008 ▶); molecular graphics: *ORTEP-3 for Windows* (Farrugia, 2012 ▶); software used to prepare material for publication: *SHELXL97*.

## Supplementary Material

Crystal structure: contains datablock(s) I, global. DOI: 10.1107/S1600536814011787/tk5317sup1.cif

Structure factors: contains datablock(s) I. DOI: 10.1107/S1600536814011787/tk5317Isup2.hkl

Supporting information file. DOI: 10.1107/S1600536814011787/tk5317Isup3.cml

CCDC reference: 1004507

Additional supporting information:  crystallographic information; 3D view; checkCIF report

## Figures and Tables

**Fig. 1.**
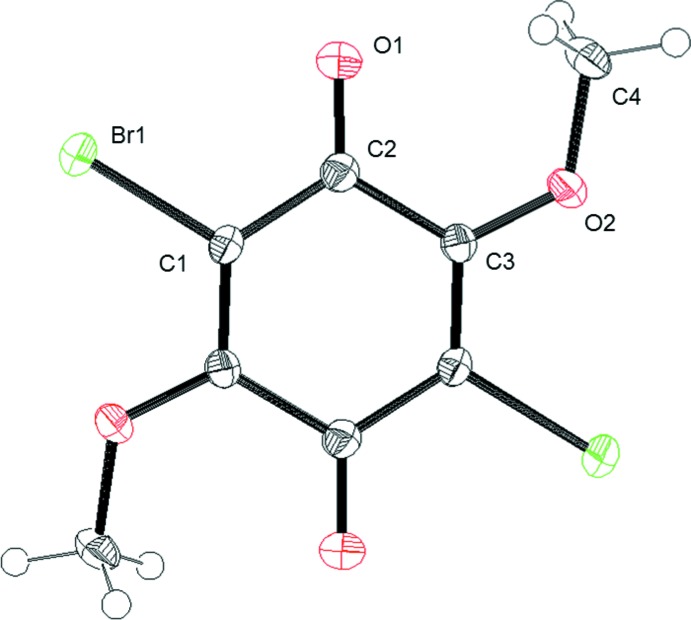
The molecular structure of 2,5-dibromo-3,6-dimethoxy-2,5-cyclohexadiene-1,4-dione (symmetry operation i = -x, 1 - y, 2 - z). Displacement ellipsoids are drawn at the 50% probability level.

## supplementary crystallographic information

### 2. Structural commentary

Embelin (2,5-di­hydroxy-3-undecyl­cyclo­hexa-2,5-diene-1,4-dione) and its derivatives possess great biological potential (Viault *et al.*, 2013). Over the years, several synthetic strategies have been developed to prepare analogues of Embelin (Khan & Driscoll, 1976; Tatsuta *et al.*, 2001; Kasahara & Kondo, 2006; Gan *et al.*, 2009; Viault *et al.*, 2011), and among the precursors used to synthesize these Embelin derivatives, 2,5-di­bromo-3,6-di­meth­oxy­cyclo­hexa-2,5-diene-1,4-dione (C_8_H_6_Br_2_O_4_) is often encountered. Moreover, such 2,5-di­hydroxy-1,4-benzo­quinones are commonly used as building blocks to generate metalla-assemblies (Therrien, 2009; Gupta *et al.*, 2014), which explains our inter­est in the title compound. The molecular structure is presented in Fig. 1.

In the solid-state, the molecule, which sits about an inversion centre, is planar with the methyl groups being only ±0.321 (4) Å out of this plane (the plane defined by the di­bromo­benzo­quinone unit including the two O atoms of the meth­oxy groups has a r.m.s. deviation of 0.0358 Å). The electron delocalization within the cyclo­hexadiene core is reflected in the C—C bonds, which show inter­mediate values instead of the typical C—C and C═C bond distances. A similar pattern of C—C bond distances was observed in the analogous compound bromanilic acid (Robl, 1987) and other substituted 2,5-cyclo­hexadiene-1,4-dione derivatives (Nakatsuji *et al.*, 2009).

### 3. Supra­molecular features

In the crystal packing Br···O(meth­oxy) halogen bonds [3.2407 (19) Å] connect molecules into supra­molecular layers parallel to (101).

### 5. Synthesis and crystallization

2,5-Di­bromo-3,6-di­meth­oxy­cyclo­hexa-2,5-diene-1,4-dione was prepared according to a published method (Viault *et al.*, 2011). Crystals were obtained by slow evaporation of an ethyl acetate solution containing the title compound.

### 6. Refinement

Hydrogen atoms were included in calculated positions and treated as riding atoms, with C—H = 0.96 Å, and with *U*_iso_(H) = 1.5*U*_eq_(C).

### Crystal data

**Table:**

C_8_H_6_Br_2_O_4_	*F*(000) = 312
*M**_r_* = 325.95	*D*_x_ = 2.276 Mg m^−^^3^
Monoclinic, *P*2_1_/*n*	Mo *K*α radiation, λ = 0.71073 Å
Hall symbol: -P 2yn	Cell parameters from 7998 reflections
*a* = 9.4456 (9) Å	θ = 2.4–25.9°
*b* = 5.4877 (3) Å	µ = 8.50 mm^−^^1^
*c* = 10.0341 (9) Å	*T* = 173 K
β = 113.846 (7)°	Block, red
*V* = 475.71 (7) Å^3^	0.23 × 0.21 × 0.20 mm
*Z* = 2	

### Data collection

**Table:**

Stoe IPDS diffractometer	1284 independent reflections
Radiation source: fine-focus sealed tube	1144 reflections with *I* > 2σ(*I*)
Graphite monochromator	*R*_int_ = 0.071
Detector resolution: 0 pixels mm^-1^	θ_max_ = 29.2°, θ_min_ = 2.5°
φ oscillation scans	*h* = −12→12
Absorption correction: part of the refinement model (Δ*F*) (*DIFABS*; Walker & Stuart, 1983)	*k* = −7→7
*T*_min_ = 0.246, *T*_max_ = 0.704	*l* = −13→13
8772 measured reflections	

### Refinement

**Table:**

Refinement on *F*^2^	Primary atom site location: structure-invariant direct methods
Least-squares matrix: full	Secondary atom site location: difference Fourier map
*R*[*F*^2^ > 2σ(*F*^2^)] = 0.028	Hydrogen site location: inferred from neighbouring sites
*wR*(*F*^2^) = 0.067	H-atom parameters constrained
*S* = 1.04	*w* = 1/[σ^2^(*F*_o_^2^) + (0.0431*P*)^2^] where *P* = (*F*_o_^2^ + 2*F*_c_^2^)/3
1284 reflections	(Δ/σ)_max_ < 0.001
65 parameters	Δρ_max_ = 0.86 e Å^−^^3^
0 restraints	Δρ_min_ = −0.98 e Å^−^^3^

### Special details

**Table:**

Experimental. A crystal was mounted at 173 K on a Stoe Image Plate Diffraction System (Stoe & Cie, 2000) using Mo *K*α graphite monochromated radiation. Image plate distance 100 mm, φ oscillation scans 0 - 180°, step Δφ = 1.2°, 3 minutes per frame.
Geometry. All e.s.d.'s (except the e.s.d. in the dihedral angle between two l.s. planes) are estimated using the full covariance matrix. The cell e.s.d.'s are taken into account individually in the estimation of e.s.d.'s in distances, angles and torsion angles; correlations between e.s.d.'s in cell parameters are only used when they are defined by crystal symmetry. An approximate (isotropic) treatment of cell e.s.d.'s is used for estimating e.s.d.'s involving l.s. planes.
Refinement. Refinement of *F*^2^ against ALL reflections. The weighted *R*-factor *wR* and goodness of fit *S* are based on *F*^2^, conventional *R*-factors *R* are based on *F*, with *F* set to zero for negative *F*^2^. The threshold expression of *F*^2^ > σ(*F*^2^) is used only for calculating *R*-factors(gt) *etc*. and is not relevant to the choice of reflections for refinement. *R*-factors based on *F*^2^ are statistically about twice as large as those based on *F*, and *R*- factors based on ALL data will be even larger.

### Fractional atomic coordinates and isotropic or equivalent isotropic displacement parameters (Å^2^)

**Table:**

	*x*	*y*	*z*	*U*_iso_*/*U*_eq_	
Br1	0.14128 (3)	0.21073 (5)	0.79296 (3)	0.02303 (10)	
C2	−0.0827 (3)	0.2940 (4)	0.9112 (3)	0.0197 (4)	
C4	−0.3943 (3)	0.2112 (5)	0.9080 (3)	0.0253 (5)	
H4A	−0.4045	0.2467	0.8109	0.038*	
H4B	−0.4925	0.2333	0.9138	0.038*	
H4C	−0.3608	0.0457	0.9320	0.038*	
O2	−0.2815 (2)	0.3740 (4)	1.00971 (19)	0.0262 (4)	
O1	−0.1483 (2)	0.1143 (4)	0.8426 (2)	0.0332 (4)	
C1	0.0654 (3)	0.3806 (4)	0.9134 (2)	0.0176 (4)	
C3	−0.1485 (2)	0.4344 (4)	1.0024 (2)	0.0174 (4)	

### Atomic displacement parameters (Å^2^)

**Table:**

	*U*^11^	*U*^22^	*U*^33^	*U*^12^	*U*^13^	*U*^23^
Br1	0.02309 (14)	0.02542 (15)	0.02416 (15)	0.00012 (9)	0.01326 (10)	−0.00545 (8)
C2	0.0212 (11)	0.0197 (10)	0.0193 (11)	−0.0009 (8)	0.0093 (9)	−0.0011 (8)
C4	0.0178 (10)	0.0290 (12)	0.0266 (12)	−0.0072 (9)	0.0064 (9)	−0.0016 (9)
O2	0.0216 (8)	0.0326 (9)	0.0283 (9)	−0.0113 (7)	0.0143 (7)	−0.0098 (8)
O1	0.0311 (10)	0.0297 (10)	0.0443 (11)	−0.0119 (8)	0.0209 (9)	−0.0171 (9)
C1	0.0185 (10)	0.0190 (10)	0.0169 (9)	0.0013 (8)	0.0090 (8)	−0.0011 (8)
C3	0.0176 (9)	0.0184 (10)	0.0177 (9)	−0.0004 (8)	0.0086 (8)	0.0012 (7)

### Geometric parameters (Å, º)

**Table:**

Br1—C1	1.882 (2)	C4—H4B	0.9600
C2—O1	1.219 (3)	C4—H4C	0.9600
C2—C1	1.469 (3)	O2—C3	1.330 (3)
C2—C3	1.509 (3)	C1—C3^i^	1.351 (3)
C4—O2	1.448 (3)	C3—C1^i^	1.351 (3)
C4—H4A	0.9600		
			
O1—C2—C1	122.3 (2)	H4B—C4—H4C	109.5
O1—C2—C3	121.0 (2)	C3—O2—C4	123.90 (19)
C1—C2—C3	116.68 (19)	C3^i^—C1—C2	124.14 (19)
O2—C4—H4A	109.5	C3^i^—C1—Br1	119.87 (16)
O2—C4—H4B	109.5	C2—C1—Br1	115.96 (16)
H4A—C4—H4B	109.5	O2—C3—C1^i^	118.4 (2)
O2—C4—H4C	109.5	O2—C3—C2	122.4 (2)
H4A—C4—H4C	109.5	C1^i^—C3—C2	119.05 (19)
			
O1—C2—C1—C3^i^	−174.0 (2)	C4—O2—C3—C2	−15.5 (4)
C3—C2—C1—C3^i^	4.2 (4)	O1—C2—C3—O2	−1.7 (4)
O1—C2—C1—Br1	4.2 (3)	C1—C2—C3—O2	−179.9 (2)
C3—C2—C1—Br1	−177.59 (16)	O1—C2—C3—C1^i^	174.3 (2)
C4—O2—C3—C1^i^	168.5 (2)	C1—C2—C3—C1^i^	−4.0 (4)

Symmetry code: (i) −*x*, −*y*+1, −*z*+2.
